# Metal-free C_60_/CNTs/g-C_3_N_4_ ternary heterostructures: synthesis and enhanced visible-light-driven photocatalytic performance

**DOI:** 10.1098/rsos.172290

**Published:** 2018-05-16

**Authors:** Xue Lin, Rui Zhao, Yang Xi, Xiangyu Li, Junyou Shi, Ning Yan

**Affiliations:** College of Forestry, Beihua University, 3999 Binjiang Road, Jilin 132013, People's Republic of China

**Keywords:** C_60_/CNTs/g-C_3_N_4_, nanoheterostructures, photocatalysis, visible light

## Abstract

A metal-free C_60_/CNTs/g-C_3_N_4_ nanoheterostructure with excellent visible-light photocatalysis for rhodamine B (Rh B) degradation has been reported. Via a convenient low-temperature solution-phase method, g-C_3_N_4_ nanosheets can serve as substrate for dispersion of C_60_/CNTs. The loading of C_60_/CNTs onto g-C_3_N_4_ nanosheets surfaces significantly enhanced visible-light-driven photocatalytic activity of g-C_3_N_4_ catalyst, for oxidation of organic pollutant (Rh B, 100%). Excellent photocatalytic properties of C_60_/CNTs/g-C_3_N_4_ can be predominantly attributed to the intimate interfacial contact among constructing compounds, increased specific surface area and enhanced light adsorption efficiency resulted from C_60_/CNTs carbon materials. Particularly, the synergistic heterostructure interaction remarkably hinders the electron–hole pairs recombination, giving rise to significantly enhanced photocatalytic performance of C_60_/CNTs/g-C_3_N_4_ in comparison with other counterparts.

## 1. Introduction

Photocatalysis employing semiconductor materials has been considered as a promising method for widespread solar energy utilization and environmental remediation [1–3]. During the past few decades, many research works on photocatalysis field are mainly focused on the development of metal-oxide-based semiconductors, owing to their high thermal and chemical stability [4,5]. Nevertheless, the wide band gap and low quantum efficiency of some metal-oxide-based semiconductors significantly limit their potential applications as visible-light photocatalysts. Therefore, it is highly desirable to explore non-metal oxide photocatalysts for a wide range of prospective applications for visible-light photocatalysis [6].

Graphitic carbon nitride (g-C_3_N_4_), a metal-free polymeric photocatalyst, is considered as a multifunctional catalyst for photo/electrocatalytic water splitting, CO_2_ reduction and organic pollutant degradation due to its appealing electronic structure, excellent chemical and thermal stabilities, low cost and environment benignity [7–9]. Nevertheless, g-C_3_N_4_ has inherently low electronic conductivity and surface area, restricting its potential applications [10]. Construction of heterostructures by combining g-C_3_N_4_ with semiconductor materials has been shown to be an efficient method to enhance the photocatalytic efficiency of g-C_3_N_4_ [11–13]. Recently, carbonaceous materials such as activated carbon (AC), fullerene (C_60_), carbon nanotubes (CNTs) and graphene (GE) with the superiorities of large surface area, good electrical conductivity and stability, and non-toxicity have shown an ability to enhance the visible-light photocatalytic performance of g-C_3_N_4_ [14–16]. For example, fullerene (C_60_) acting as electron acceptor might suppress the recombination of photo-generated electrons and holes in C_60_/g-C_3_N_4_ composite, thus improving the photocatalytic efficiency [16]. In addition, Xu *et al*. have synthesized CNTs modified g-C_3_N_4_ (CNTs/g-C_3_N_4_) composites via a hydrothermal method. The as-prepared CNTs/g-C_3_N_4_ displayed higher photocatalytic activity for degradation of methylene blue (MB) than that of single g-C_3_N_4_ [17]. The aforementioned results suggest that g-C_3_N_4_ coupled with carbonaceous materials would greatly enhance its photocatalytic activity. Owing to the excellent light absorption ability and good electron conductivity of carbon materials, it can be speculated that concurrent decoration of g-C_3_N_4_ with CNTs and C_60_ would significantly enhance the photocatalytic activities of g-C_3_N_4_. However, to the best of our knowledge, there is no reported investigation focused on the photocatalytic activity of C_60_/CNTs/g-C_3_N_4_ composite, and systematic study on the photocatalysis mechanism has not yet been reported.

Herein, we developed a homologous, metal-free, g-C_3_N_4_-based catalyst for photocatalytic application. It has been revealed that photocatalytic performance of g-C_3_N_4_ can be conspicuously improved by loading C_60_/CNTs towards the degradation of rhodamine B (Rh B) under visible-light illumination. The loading of C_60_/CNTs contributes to the significantly enhanced photocatalytic activity of C_60_/CNTs/g-C_3_N_4_ nanoheterostructure. Furthermore, the underlying photocatalysis mechanism was studied. The regeneration and reusability of the C_60_/CNTs/g-C_3_N_4_ nanocomposite was also investigated.

## 2. Material and methods

### 2.1. Preparation of photocatalysts

#### 2.1.1. Preparation of C_60_/CNTs

C_60_-decorated single-walled carbon nanotubes (C_60_/CNTs) was prepared by using the Zhang’ method [16]: SWCNTs (50 mg) and C_60_ (50 mg) were dispersed by ultrasonication in toluene (100 ml) for 1 h, and then stirred at room temperature for 12 h. After volatilization of the toluene, the resultant black powder was washed with ethanol several times and dried in vacuum at 80°C for 10 h (denoted as C_60_/CNTs).

#### 2.1.2. Preparation of C_60_/CNTs/g-C_3_N_4_

C_60_/CNTs/g-C_3_N_4_ ternary composite was fabricated via a facile hydrothermal method. Especially, C_60_/CNTs (0.05 g), g-C_3_N_4_ (0.05 g) powders were added into ethanol solution (15 ml). The suspension was ultrasonicated for 1 h. The mixed solution was then transferred to a 25 ml Teflon-lined stainless steel autoclave reactor and heated at 180°C for 3 h. The C_60_/CNTs/g-C_3_N_4_ sample was then collected. The C_60_/g-C_3_N_4_ and CNTs/g-C_3_N_4_ binary composite was synthesized through a similar method.

### 2.2. Characterization of photocatalysts

The morphology of the samples was studied by SEM (JSM-7800F) and TEM (JEM-2100F). The crystal structure of the samples was tested with XRD (Rigaku, D/max 2500) equipped with Kα radiation. Fourier transform infrared (FTIR) spectra of the samples were recorded on infrared spectrophotometer (America Perkin Elmer, Spectrum One). UV–vis diffuse reflection spectroscopy (DRS) was performed on a scan UV–vis spectrophotometer (SHIMADZU, UV-2550). The X-ray photoelectron spectroscopy (XPS, VGScientific) using 300 W Al Kα radiation as the excitation source was employed for the surface analysis of the prepared C_60_/CNTs/g-C_3_N_4_ nanocomposite. The surface areas were studied by the nitrogen adsorption Brunauer–Emmett–Teller (BET) method (BET/BJH Surface Area, 3H-2000PS1). The photoluminescence (PL) spectra of the photocatalysts were measured on a F4500 (Hitachi, Japan) photoluminescence detector with an excitation wavelength of 325 nm.

### 2.3. Photocatalytic activities studies

Photocatalytic activities of the photocatalysts were evaluated by testing the degradation efficiencies of Rh B, using a Xe lamp (500 W), which was equipped with a UV irradiation source filter (*λ* > 420 nm). In a standard photocatalytic experiment, the photocatalyst (50 mg) was dispersed in a mixed aqueous solution of Rh B (50 ml, 0.01 mmol l^−1^), and the suspension was stirred for 40 min in the dark to ensure that it reached the adsorption–desorption equilibrium. Then, the suspension was illuminated by visible light. At 5 min intervals, a 4.0 ml of the suspension was withdrawn, and centrifuged to remove the catalyst. The concentration of Rh B was analysed by UV–vis spectrophotometry.

## 3. Results and discussion

**Figure 1.**
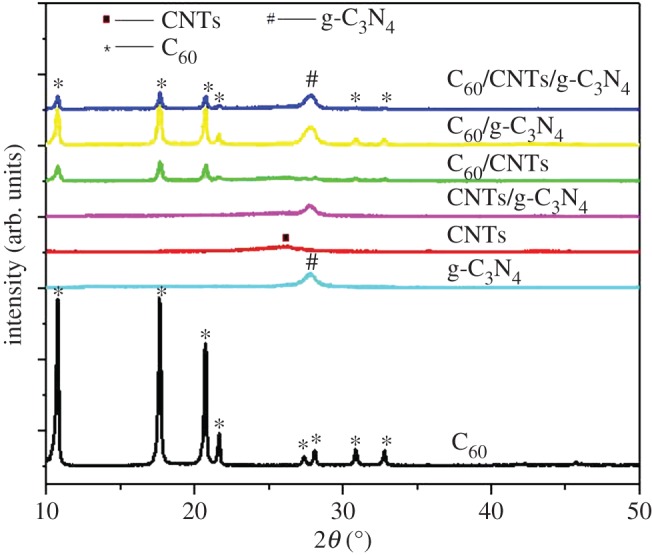
XRD patterns of the as-prepared samples.

**Figure 2.**
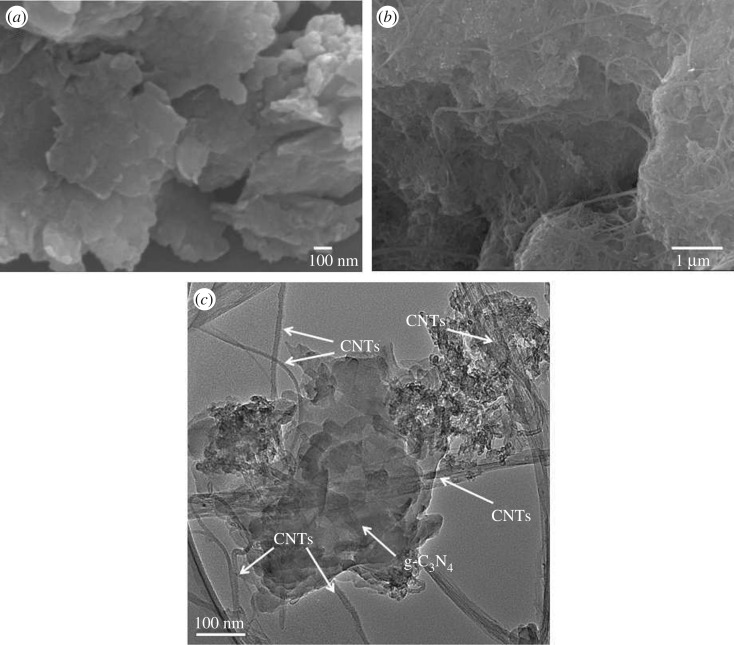
SEM images of the as-prepared samples: (*a*) g-C_3_N_4_, (*b*) C_60_/CNTs/g-C_3_N_4_, (*c*) TEM image of the C_60_/CNTs/g-C_3_N_4_.

The phase and structure of photocatalysts were studied by XRD studies. As shown in figure 1, the diffraction peaks at 27.5° for g-C_3_N_4_ are detected in the pattern of g-C_3_N_4_. The characteristic peaks of the C_60_/g-C_3_N_4_ hybrid were respectively attributed to g-C_3_N_4_ (27.5°) [18], and C_60_ (10.7°, 17.7°, 20.7° and 21.7°), indicating the successful formation of the homogeneous hybrid structure [15]. Alternatively, there is also no difference observed between the C_60_/g-C_3_N_4_ and C_60_/CNTs/g-C_3_N_4_, probably due to low loading amount and the weak diffraction of CNTs [19,20]. Figure 2 depicts the microstructures of C_60_/CNTs/g-C_3_N_4_ composite. As shown in figure 2*a*, g-C_3_N_4_ has layered structure composed of tightly stacked g-C_3_N_4_ nanosheets with sizes of the thickness of tens of nanometres. As shown in figure 2*b,c*, some CNTs with an average diameter of around 10 nm can also be observed, which are attached on the sheet-like g-C_3_N_4_. C_60_ molecules with a diameter of approximately 0.7 nm are too small to be directly resolved by TEM observation, which is similar to the previous report [16].

**Figure 3.**
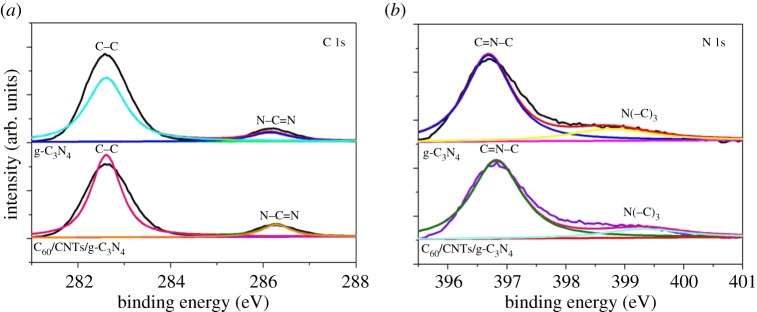
XPS spectra of the as-obtained samples: (*a*) C 1s and (*b*) N 1s.

The XPS measurement was performed to determine the chemical composition and valence state of various species. The C 1s (figure 3*a*) high-resolution spectrum in C_60_/CNTs/g-C_3_N_4_ nanocomposite and g-C_3_N_4_ both have two distinct peaks at around 282.6 and 286.2 eV. The peak at 286.2 eV is identified as sp^2^-bonded carbon (N–C=N). The peak located at 284.6 eV could be assigned to adventitious carbon, C–C bond from the C_60_ and graphitic carbon [16,21]. The high-resolution XPS spectrum for N 1s in the g-C_3_N_4_ could be fitted into two peaks at 396.7 and 399.0 eV (figure 3*b*), corresponding to the pyridinic N species in triazine rings (C–N=C) and tertiary N species or N atoms bonded with H atoms [22]. In the case of C_60_/CNTs/g-C_3_N_4_, the binding energy of N 1s shifted about 0.2 eV, toward higher binding energies, which was caused by the strong interaction between the CNTs and the N atoms in the g-C_3_N_4_ [23].

**Figure 4.**
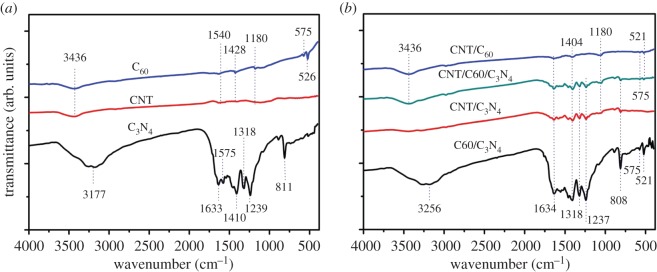
(*a*,*b*) FTIR of the as-prepared samples.

FTIR spectroscopy was further applied to identify the existence of C_60_ and g-C_3_N_4_ in the C_60_/CNTs/g-C_3_N_4_ composite (figure 4*a,b*). For pure g-C_3_N_4_ (figure 4*a*), the peaks at 1239, 1318, 1410, 1575 and 1633 cm^−1^ feature typical aromatic C–N heterocycle stretches, and the peaks at 811 cm^−1^ are corresponding to the tri-*s*-trizine units of g-C_3_N_4_ [23]. It also can be seen in figure 4*a* that for pure C_60_, the bands at 526, 575, 1180, 1428 and 1540 cm^−1^ are attributed to the internal modes of the C_60_ molecule [16]. For C_60_/CNTs/g-C_3_N_4_, both the characteristic peaks of g-C_3_N_4_ and C_60_ can be clearly observed, identifying the successful incorporation of C_60_ and g-C_3_N_4_ in the ternary composite (figure 4*b*).

**Figure 5.**
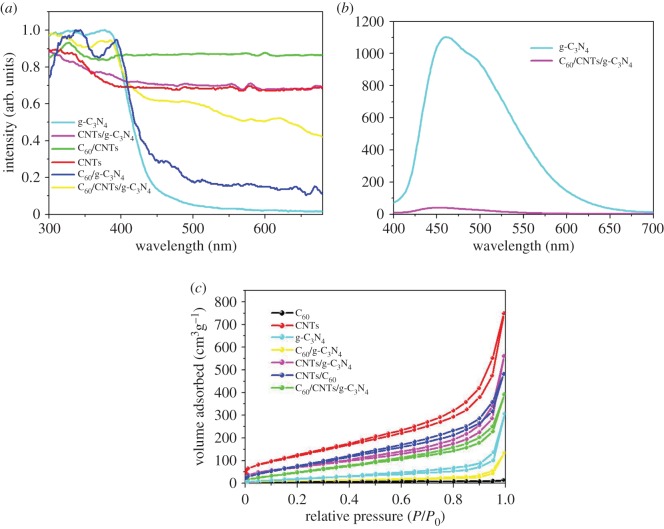
(*a*) DRS of the as-prepared samples. (*b*) PL spectra of the as-synthesized g-C_3_N_4_ and C_60_/CNTs/g-C_3_N_4_. (*c*) N_2_ adsorption–desorption isotherm curves of the as-prepared samples.

Figure 5*a* shows the UV–vis diffuse reflectance spectra (DRS) of the as-fabricated samples. As can be seen, g-C_3_N_4_ demonstrated strong absorption ability in visible region. Visible-light harvesting ability of C_60_/g-C_3_N_4_ and CNTs/g-C_3_N_4_ was substantially enhanced with adsorption edge red shift to 500 and 550 nm, respectively. The result shows that integration of C_60_ or CNTs in the nanocomposites could improve the light harvesting efficiency of g-C_3_N_4_. In addition, the loading of C_60_/CNTs in the g-C_3_N_4_ leads to a further enhancement in the absorption edge (approx. 530 nm), which reveals the key role of CNTs for promoting the light harvesting and thus leading to the excitation of more electrons over the C_60_/CNTs/g-C_3_N_4_ nanocomposite under visible light [23].

The PL spectrum was employed to study the charge-carriers separation/recombination behaviour of photocatalysts. As shown in figure 5*b*, the PL emission peaks at around 460 nm for the samples reveals that the π-conjugated system of g-C_3_N_4_ was not destroyed by the modification treatment [24]. The decrease of PL intensity of C_60_/CNTs/g-C_3_N_4_ ternary nanocomposites in comparison with pure g-C_3_N_4_ shows that recombination of photo-generated electrons and holes was inhibited with the loading of C_60_/CNTs.

Full nitrogen adsorption isotherms of the as-fabricated samples were studied to gain the information about the specific surface area, as shown in figure 5*c*. The textural parameters of the photocatalysts are summarized in table 1. As can be seen, the loading of C_60_ onto g-C_3_N_4_ or CNTs could lead to a significant decrease in the BET-specific surface, which may be due to the formation of C_60_ clusters in the mesopores of g-C_3_N_4_ or CNTs. It should be noted that the loading of CNTs onto g-C_3_N_4_ could lead to a significant increase in the BET-specific surface. In addition, the surface area of C_60_/CNTs/g-C_3_N_4_ composite is obviously smaller than those of CNTs/g-C_3_N_4_, which may be due to the addition of C_60_.

**Table 1.** BET-specific surface area of the as-prepared samples.

photocatalysts	BET-specific surface area (m^2^ g^−1^)	photocatalysts	BET-specific surface area (m^2^ g^−1^)
CNTs	452.023	C_60_/g-C_3_N_4_	30.231
C_60_	177.399	CNTs/g-C_3_N_4_	254.202
g-C_3_N_4_	72.984	C_60_/CNTs/g-C_3_N_4_	181.227
C_60_/CNTs	277.847		

**Figure 6.**
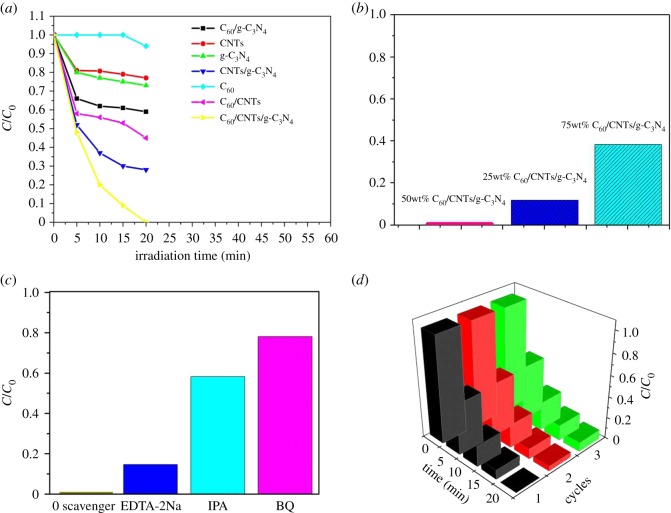
(*a*) Photodegradation efficiencies of Rh B as a function of irradiation time for different photocatalysts. (*b*) Photodegradation efficiencies of Rh B as a function of irradiation time for different photocatalysts. (*c*) Trapping experiment of active species during the photocatalytic degradation of Rh B over C_60_/CNTs/g-C_3_N_4_ nanocomposites under visible-light irradiation. (*d*) Cycling runs for the photocatalytic degradation of Rh B over the ternary nanocomposites under visible-light irradiation.

Figure 6*a* displays the photocatalytic activities of bare g-C_3_N_4_, C_60_, CNTs, C_60_/CNTs, CNTs/g-C_3_N_4_, C_60_/g-C_3_N_4_ and C_60_/CNTs/g-C_3_N_4_ for the degradation of Rh B solution. Only 27.0%, 6.0% and 23.0% of Rh B were removed by pure g-C_3_N_4_, C_60_, CNTs, respectively, after 60 min irradiation, while 41.0%, 55.0% of Rh B by C_60_/g-C_3_N_4_ and CNTs/g-C_3_N_4_, approximately. It means that C_60_ or CNTs loading is beneficial for the improvement of photocatalytic activity of the composite sample. Meanwhile, the photocatalytic efficiency of C_60_/CNTs also rose to 72.0%. The photocatalytic efficiency of C_60_/CNTs/g-C_3_N_4_ reached 100%, which is remarkably faster than the rate of bare g-C_3_N_4_, C_60_, CNTs, C_60_/CNTs, CNTs/g-C_3_N4 and C_60_/g-C_3_N_4_. It is due to that efficient heterostructure interface among three components that can restrain the recombination of photo-induced charges effectively [25]. In addition, the C_60_/CNTs coating can improve the visible-light absorption efficiency (figure 5*a*) and BET-specific surface (table 1) of the photocatalyst, which are both beneficial for the ternary composite to photolyse Rh B. In addition, the effect of C_60_ amount on the photocatalytic activity of the composites was also investigated (figure 6*b*). Furthermore, the highest degradation rate was obtained from C_60_(50_wt_%)/CNTs/g-C_3_N_4_ sample with almost 100% of Rh B removal. This increase may be attributed to the capturing of electrons by the deposited C_60_ to hinder the recombination of hole–electron pairs [26,27], whereas the decrease may result from the blocking of visible light by the over-deposited C_60_. In order to further understand the photocatalytic mechanism of the ternary nanocomposite, the trapping experiment of active species was also investigated. As shown in figure 6*c*, isopropanol (IPA), triethanolamine (TEOA) and *p*-benzoquinone (BQ) were, respectively, introduced as the scavengers of hydroxyl radicals (·OH), holes (h^+^) and superoxide radicals (·O^2−^) to examine the effects of reactive species on the photocatalytic degradation of Rh B. It can be seen that BQ and IPA led to a remarkable suppression of the degradation rate of Rh B, whereas EDTA-2Na exhibited a weaker restraining effect on the degradation rate. The results confirmed that ·OH and ·O^2−^ play a more important role than h^+^ in the photocatalytic degradation of Rh B.

**Figure 7.**
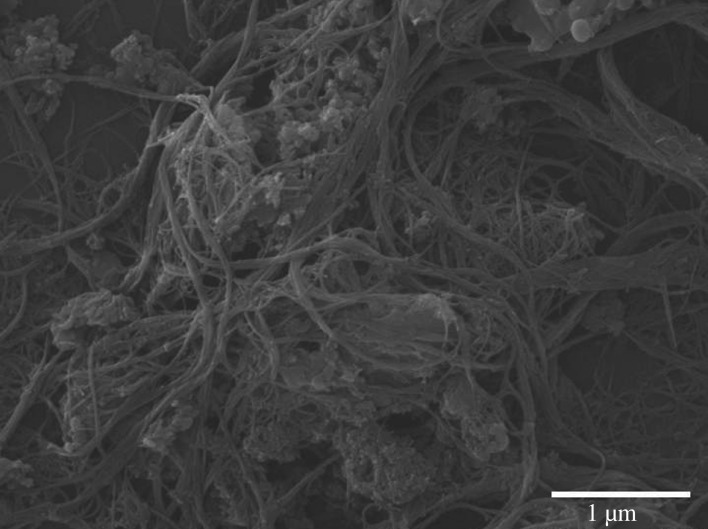
SEM image of C_60_/CNTs/g-C_3_N_4_ composite after photocatalysis.

The stability and reusability of C_60_/CNTs/g-C_3_N_4_ for photocatalytic degradation of Rh B were evaluated by repeating the photocatalytic experiments under the same conditions for three cycles. It is found that no obvious deactivation in the photocatalytic activity is detected for the C_60_/CNTs/g-C_3_N_4_ composite after continuous visible-light irradiation (figure 6*d*). Figure 7 shows the SEM image of the C_60_/CNTs/g-C_3_N_4_ sample after the photocatalytic reaction. After four catalytic runs, the secondary C_60_/CNTs/g-C_3_N_4_ nanostructures were still very intact, clearly showing their stabilities. Thus, it represents a promising kind of composite catalyst for Rh B degradation under visible light.

**Figure 8.**
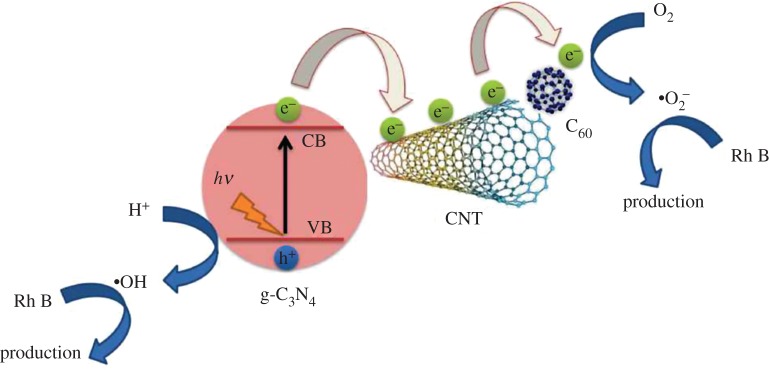
Schematic diagram of the separation and transfer of photo-generated charges in the composite photocatalyst under visible-light irradiation.

Based on the above analysis, a schematic illustration of the photocatalysis mechanism of the C_60_/CNTs/g-C_3_N_4_ is displayed in figure 8. Under visible-light illumination, photo-induced electrons are excited from the valence band (VB) of g-C_3_N_4_ to the conduction band (CB), resulting in electron–hole pairs. The CB of g-C_3_N_4_ (–1.12 eV versus NHE) is less negative than that of the CNTs (–0.3 eV versus NHE) or C_60_ (−0.2 eV versus NHE) [28,29]; therefore, photoelectrons from the CB of g-C_3_N_4_ are captured by the CNTs or C_60_. As a result, the photoactivities of CNTs/g-C_3_N_4_, C_60_/g-C_3_N_4_ have been enhanced compared with the bare g-C_3_N_4_. For the C_60_/CNTs/g-C_3_N_4_ nanocomposite, there are two possible ways for the transformation of photo-generated charges: (i) the electrons may firstly transfer to C_60_ and then to CNTs, due to that, as the strong electron acceptor, the fullerenes can capture the electrons, which are spread and moved along the CNTs and fullerenes, thus facilitating the photo-generated carrier separation; (ii) the electrons in the CB of g-C_3_N_4_ may firstly transfer to CNTs, and then to C_60_. Consequently, the carrier recombination can be effectively inhibited. In this way, the excited electrons participate in the reduction of O_2_ to ·O^2−^. Subsequently, the ·O^2−^ and ·OH oxidize the Rh B to CO_2_ and H_2_O.

## 4. Conclusion

In summary, we have demonstrated that C_60_/CNTs/g-C_3_N_4_ composite was synthesized by a simple hydrothermal technology. The results showed that the C_60_/CNTs/g-C_3_N_4_ nanocomposite exhibited the best photocatalytic activity under visible light, which is almost five times higher than that of pure g-C_3_N_4_. It shows that the ternary heterostructure can effectively hinder the recombination of the photo-generated electron–hole pairs, and thus improve the photocatalytic performance of the C_60_/CNTs/g-C_3_N_4_ nanocomposite. In this work, the enhancement of photocatalytic activity of C_60_/CNTs/g-C_3_N_4_ could be achieved via the loading C_60_/CNTs, which may provide a promising class of photocatalyst candidates for photocatalytic application.

## Supplementary Material

Supplementary data

## Data accessibility

The datasets supporting this article have been uploaded as the electronic supplementary material.
